# Systemic Autoimmune Diseases

**DOI:** 10.1155/2013/728574

**Published:** 2013-12-01

**Authors:** Guixiu Shi, Jianying Zhang, Zhixin (Jason) Zhang, Xuan Zhang

**Affiliations:** ^1^Department of Rheumatology and Clinical Immunology, The First Hospital of Xiamen University, Xiamen 361003, China; ^2^Department of Biological Sciences, University of Texas at El Paso, 500 West University Avenue, El Paso, TX 79968, USA; ^3^Microbiology, College of Medicine University of Nebraska Medical Center, Omaha, NE 68198-7660, USA; ^4^Department of Rheumatology, Peking Union Medical College Hospital, Peking Union Medical College, Chinese Academy of Medical Sciences, Beijing 100730, China

Systemic autoimmune diseases are a broad range of related diseases characterized by dysregulation of immune system which give rise to activation of immune cells to attack autoantigens and resulted in inappropriate inflammation and multitissue damages. They are a fascinating but poorly understood group of diseases, ranging from the commonly seen rheumatoid arthritis (RA) and systemic lupus erythematosus (SLE) to the relatively rare systemic sclerosis [1]. The mechanism of pathogenesis of systemic autoimmune diseases is still not very clear. It is now considered that genetic factors, infection, endocrine, and environmental exposure are involved in the pathogenesis of these diseases [2–4]. There is no treatment strategy to cure this kind of disease at present which gives rise to the needs of long-term lasting treatment, making systemic autoimmune diseases be a mounting public health concern for the foreseeable future. Vital organs such as lung and kidney involvement in systemic autoimmune diseases are common and always presented in a progressive pattern with limited treatment strategy, making them be one of the most common causes of death in patients [5].

Based on this background, we assembled this special issue for a better understanding of systemic autoimmune diseases, on aspects of mechanisms of pathogenesis, diagnosis, and treatment, including papers ranging from the basic researches to clinical researches and reviews about systemic autoimmune diseases.

Studies on the basic research of systemic autoimmune disease in this issue provided us new insights into the mechanism of the pathogenesis of systemic autoimmune disease. The role of HMGB1 in the T-cell DNA demethylation was discussed in the paper of Y. Li et al. SNPs with strong RA association signal in the British were analyzed in Han Chinese by H. Li et al., and the methylation status of miR-124a loci in synovial tissues of RA patients was analyzed by Q. Zhou et al. indicating the epigenetic factor in the pathogenesis of RA. Expression of microRNA-155 was studied by L. Long et al. in RA patients. IL-33 status was tested in RA patients by S. Tang et al. D. Lorton et al. and Y. Du et al. indicated the possible role of beta2-adrenergic receptors (*β*2-AR) and p53 apoptosis effector related to PMP-22(Perp) in the pathogenesis of RA, respectively. It has long been demonstrated that *γδ* T cells play important roles in the development of autoimmune diseases; the precise role of *γδ* T cells in the pathogenesis of SLE was studied by Z. Lu et al. Z. Gu et al. discussed the role of p53/p21 pathway in the pathogenesis of SLE. X. Gan et al. demonstrated the role of GITR and GITRL in the primary Sjögren's syndrome. The expression of IL-6 and its clinical significance in patients with dermatomyositis was discussed by M. Yang et al. L. Estrada-Capetillo et al. found that DCs from patients with rheumatic inflammatory disease show an aberrant function that may have an important role in the pathogenesis. S. Stratakis et al. studied the mechanisms underlying this beneficial effect of rapamycin in passive and active Heymann nephritis (HN).

Clinical researches and studies included in this issue provided us several useful clinical clues in the diagnosis, treatment, and prediction of some of systemic autoimmune diseases. L. Hongyan et al. studied the clinical and pathologic features in lupus nephritis with mainly IgA deposits and made a literature review about this topic. Risk factors for interstitial lung disease in patients with idiopathic inflammatory myopathy were analyzed by X. Cen et al. J. Chen et al. defined high resolution chest CT (HRCT) and pulmonary function test (PFT) abnormalities capable of identifying asymptomatic, preclinical RA-ILD. L. Pan et al. made a retrospective study to compare the characteristics of connective tissue disease-associated interstitial lung diseases, undifferentiated connective tissue disease-associated interstitial lung diseases, and idiopathic pulmonary fibrosis. Relationship between Brachial-ankle pulse wave velocity (baPWV) and its associated risk factors in Chinese patients with RA was analyzed by P. Li et al. P. Žigon et al. studied the diagnostic value of antiphosphatidylserine/prothrombin antibodies in systemic autoimmune disease. The correlations of disease activity, socioeconomic status, quality of life, and depression/anxiety in Chinese SLE patients were studied by B. Shen et al. J. Li et al. made a systematic review on efficacy and safety of Iguratimod for the treatment of rheumatoid arthritis.

Review papers also cover many aspects about systemic autoimmune disease. Advances in the knowledge of costimulatory pathways and their role in SLE were discussed by N. Y. Kow and A. Mak H. Draborg et al. summed up existing data about the relationship between epstein-barr virus and autoimmune disease. T. Marchetti et al. discussed obstetrical antiphospholipid syndrome from pathogenesis to the clinical and therapeutic implications. Y. f. Huang et al. summarized the immune factors involved in the pathogenesis, diagnosis, and treatment of Sjogren's syndrome. The role of IL-33 in rheumatic diseases was reviewed by L. Duan et al. T. Ito made a review on advances in the pathogenesis of autoimmune hair loss disease alopecia areata. A. W. J. M. Glaudemans reviewed the use of 18F-FDG-PET/CT for diagnosis and treatment monitoring of inflammatory and infectious diseases. The role of Fc*γ*R-mediated trogocytosis in the physiological immune system was discussed by S. Masuda et al.

This special issue covers many important aspects in the systemic autoimmune diseases ranging from novel insights into the pathogenesis of autoimmune disease and the use of newly developed diagnostic strategy in the early diagnosis of autoimmune disease to the treatment of these kinds of diseases, which will surely provide us a better understanding about systemic autoimmune disease.

*Guixiu  Shi* *Jianying  Zhang* *Zhixin  (Jason)  Zhang* *Xuan  Zhang*
